# Linking GERD and the Peptide Bombesin: A New Therapeutic Strategy to Modulate Inflammatory, Oxidative Stress and Clinical Biochemistry Parameters

**DOI:** 10.3390/antiox13091043

**Published:** 2024-08-28

**Authors:** Alessio Ardizzone, Sarah Adriana Scuderi, Lelio Crupi, Michela Campolo, Irene Paterniti, Anna Paola Capra, Emanuela Esposito

**Affiliations:** Department of Chemical, Biological, Pharmaceutical and Environmental Sciences, University of Messina, 98166 Messina, Italy; aleardizzone@unime.it (A.A.); sarahadriana.scuderi@unime.it (S.A.S.); lelio.crupi@unime.it (L.C.); campolom@unime.it (M.C.); ipaterniti@unime.it (I.P.)

**Keywords:** gastroesophageal reflux diseases (GERD), bombesin, inflammation, oxidative stress, vitamins

## Abstract

Gastroesophageal reflux disease (GERD) represents one of the most prevalent foregut illnesses, affecting a large portion of individuals worldwide. Recent research has shown that inflammatory mediators such as cytokines, chemokines, and enzymes are crucial for causing esophageal mucosa alterations in GERD patients. It seems likely that the expression of various cytokines in the esophageal mucosa also induces oxidative stress by increasing the production of reactive oxygen species (ROS) and reactive nitrogen species (RNS). As humoral agents and peptidergic neurotransmitters that may support the enterogastric axis, bombesin and its related bombesin-like peptide, GRP (gastrin releasing peptide), have not been fully investigated. Therefore, considering all these assumptions, this study aimed to evaluate the influence of bombesin in reestablishing biochemical markers linked with inflammation and oxidative/nitrosative stress in GERD pathological settings. C57BL/6 mice were alternatively overfed and fasted for 56 days to induce GERD and then treated with bombesin (0.1, 0.5, and 1 mg/kg intraperitoneally) once daily for 7 days, and omeprazole was used as the positive control. After 7 days of treatment, gastric pain and inflammatory markers were evaluated. Abdominal pain was significantly reduced following bombesin administration, which was also successful in diminishing inflammatory and oxidative/nitrosative stress markers in a manner overlapping with omeprazole. Moreover, bombesin was also able to appreciably modulate gastric pH as a result of the restoration of gastric homeostasis. Overall, these observations indicated that the upregulation of bombesin and interconnected peptides is a promising alternative approach to treat GERD patients.

## 1. Introduction

Gastroesophageal reflux disease (GERD) is a chronic gastrointestinal (GI) disorder characterized by the regurgitation of gastric contents into the esophagus with a prevalence of approximately 20% of adults in Western society [1].

Gastroesophageal reflux (GER) is a normal physiologic process that includes the involuntary flow of the stomach contents back into the esophagus [2]. Gastric juice is a harmful mixture of acid, bile, and digestive enzymes that help digest food so that it can be delivered to the small intestine. All of these components, especially acid, may damage and irritate the esophagus, causing esophagitis and reflux symptoms [3]. In physiological conditions, various mechanisms are in place to protect from reflux: the anti-reflux barrier, esophageal clearance, and esophageal mucosal resistance [2]. The anti-reflux barrier which includes also the esophagogastric junction (EGJ), is composed of the lower esophageal sphincter (LES), the angle of this, the crural diaphragm, and the pharyngoesophageal ligament [2]. Specifically, the EGJ serves as a barrier between the esophagus and the stomach to provide a mechanical and physiologic barrier to spontaneous reflux of the gastric content into the esophagus [4]. However, when these protective components are damaged, the harmful effects are intensified, leading to an increase in reflux events and exposure to more abnormal esophageal reflux. Unlike GER, which is a normal physiologic process, GERD occurs when the reflux of gastric contents causes troublesome symptoms or complications.

The pathophysiology of GERD is multifactorial [1], as several risk factors for GERD have been identified, including alcohol use, excessive body mass index (BMI), smoking, and anxiety/depression [5]. Clinically, GERD manifests with symptoms of heartburn and regurgitation [1]. However, GERD can lead to some complications, such as reflux esophagitis, which may be associated with inflammation, bleeding and ulcers due to stomach acid, esophageal stricture, and precancerous lesions [6].

GERD is caused by different mechanisms that can be intrinsic, structural, or both, leading to the disruption of the esophagogastric junction barrier and resulting in exposure of the esophagus to acidic gastric contents [1]. Specifically, it has been demonstrated that GERD is characterized by impaired esophageal mucosal integrity [7,8]. Esophageal mucosal abnormalities, such as dilated intercellular spaces (DISs), basal cell hyperplasia, and changes in epithelial thickness, have been widely described in patients with GERD [7,8].

Accordingly, some studies have reported a downregulation of tight junction (TJ)-related proteins, which generally provide structural support for barrier functions by regulating the diffusion of molecules in different gastrointestinal diseases, including GERD [9,10]. In addition to considering functional and structural abnormalities of the gastroesophageal junction, other studies have demonstrated the involvement of the inflammatory process and oxidative stress in GERD [11,12]. In the mucosa of GERD patients, high levels of inflammatory cytokines have been detected, such as interleukin-8 (IL-8), which is one of the most important neutrophil chemoattractants, IL-1β, tumor necrosis factor-α (TNF-α), and platelet activating factor (PAF), which contribute to GERD pathogenesis [13].

Currently, GERD therapy consists of proton pump inhibitors (PPIs) like omeprazole but also H2 receptor antagonists and alginates, which represent the first-line therapy for GERD, showing their effectiveness in about 80–90% of patients with erosive esophagitis [14]. However, it is critical to comprehend the full scope of PPI’s side effects as their use continue to grow, in addition to considering that many patients are not responsive to common therapies, highlighting once again how GERD is a multifaceted pathology that often requires personalized therapies.

Based on these findings, the search for new therapeutic approaches represents an important target for the management of GERD patients.

Recently, great attention was focused on the effects of bombesin, a 14-amino acid peptide that is well-known to regulate GI hormone release and GI motility in mammals [15,16]. Bombesin is a polypeptide, firstly isolated from the skin of the European fire-bellied toad *Bombina bombina*, with several physiological effects on the brain, lungs, and GI tract [15,16].

Bombesin is also involved in satiety and appetite regulation, as well as in stress-induced anorexia and pressure-induced obesity [15,17]. Despite these reports, the exact mechanisms and effects of bombesin in the GI tract are not fully understood; thus, more studies are necessary to better understand its role.

Therefore, based on these assumptions, this study aimed to assess the beneficial effects of bombesin in an in vivo murine model of GERD.

## 2. Materials and Methods

### 2.1. Materials

Bombesin was obtained from Sigma-Aldrich Company Ltd. (Milan, Italy), and detailed datasheets can be found on the manufacturer’s website. Unless otherwise noted, all materials were sourced from Sigma-Aldrich Company Ltd. (Milan, Italy). The other compounds used were of the highest commercial grade quality. All stock solutions were prepared in non-pyrogenic saline (0.9% NaCl; Baxter, Liverpool, UK).

### 2.2. Animals

CD1 adult male mice (6–8 weeks old, weighing 25 to 30 g) were sourced from Envigo, Italy, and kept in a controlled environment with a temperature of 22 ± 2 °C, relative humidity of 55 ± 15%, and a 12-h light/dark cycle. The mice were given free access to a standard diet and water. Before the experiment, the animals were quarantined for one week to ensure their suitability for the study. Animal care adhered to Italian regulations for the protection of animals used in research. The study complied with Italian law (D.M.116192), the EU Directive (2010/63/EU), and ARRIVE guidelines.

### 2.3. GERD Model and Experimental Groups

We employed a previously established model in which mice underwent alternating feeding and fasting periods over 56 days to induce GERD [12]. Once GERD was established, oral treatments were administered daily for 7 days. The chosen dose of 40 mg/kg omeprazole was based on prior research [18]. Following the seventh day of treatment, mice were euthanized through an overdose of anesthesia plus cervical dislocation. The esophagus was then surgically removed, stored, and used for biochemical and molecular biology studies. Moreover, blood, along with other biological fluids, was collected for additional biochemical analyses.

The mice were assigned randomly to the various experimental groups as indicated below:

Group 1: Sham + vehicle: mice in which GERD was not induced, then these animals were treated with the vehicle (saline) once daily for seven consecutive days (N = 8).

Group 2: GERD + vehicle: mice with GERD, then these animals were treated with vehicle (saline) once daily for seven consecutive days (N = 8).

Group 3: GERD + Omeprazole 40 mg/kg: mice with GERD, then these animals were treated with omeprazole 40 mg/kg once daily for seven consecutive days (N = 8).

Group 4: GERD + Bombesin 0.1 mg/kg: mice with GERD, then these animals were treated with bombesin 0.1 mg/kg once daily for seven consecutive days (N = 8).

Group 5: GERD + Bombesin 0.5 mg/kg: mice with GERD, then these animals were treated with bombesin 0.5 mg/kg once daily for seven consecutive days (N = 8).

Group 6: GERD + Bombesin 1 mg/kg: mice with GERD, then these animals were treated with bombesin 1 mg/kg once daily for seven consecutive days (N = 8).

### 2.4. Von Frey Test

Epigastric pain was assessed using calibrated von Frey hairs, which enabled the detection of a mechanical stimulus–response indicative of hyperalgesia associated with GERD. The mechanical threshold, expressed in grams, corresponding to the pressure that triggered an abdominal reaction (withdrawal) was automatically recorded by an electronic device. The stimulation was performed three times, and the average value was calculated as the mechanical threshold for each mouse as previously described [19,20].

Before starting the test, mice were acclimated to the behavioral chambers for 15 min before measuring the mechanical abdominal withdrawal thresholds (AWTs) using an electronic Von Frey test (dynamic plantar aesthesiometer, model 37,450; Ugo Basile, Italy), with the cutoff set at 50 g. Abdominal pain was evaluated on day 0, day 56, day 60, and day 63.

### 2.5. Gastric pH

At the end of the experiment, gastric secretions were collected using gastric lavage, as described by Crowe and colleagues [21]. In brief, following the sacrifice, the stomach was clamped at both the lower esophageal sphincter and the pyloric region to prevent emptying. Then, 200 μL of saline was injected into the stomach. The stomach was subsequently removed, and its contents were collected into a test tube. The samples were centrifuged at 13,000× *g* for 7 min, and the supernatant was aliquoted for pH measurement using a pH microelectrode XS Instruments (Modena, Italy).

### 2.6. Quantitative Reverse Transcription PCR (RT-qPCR)

Total RNA was extracted from esophageal mucosal tissue for quantitative RT-qPCR analysis using a Trizol Reagent Kit (Life Technologies, Monza, Italy), as previously indicated [22]. First-strand cDNA was synthesized from 2.0 μg of total RNA using a High-Capacity cDNA Archive Kit (Applied Biosystems, Carlsbad, CA, USA). β-actin mRNA served as an endogenous control for relative quantification. RT-qPCR was performed to evaluate the expression of MUC5AC, MUC5AB, iNOS, COX-2, Substance P, Claudin-1, Filaggrin, Zonula Occludens (ZO)-1, and Occludin using PowerUp SYBR Green Master Mix (Applied Biosystems, Carlsbad, CA, USA) on a QuantStudio 6 Flex Real-Time PCR System (Applied Biosystems, Carlsbad, CA, USA). Amplified PCR products were quantified by measuring the calculated cycle thresholds (CTs) of the target genes and β-actin mRNA. After normalization, the mean value of the target levels in the control samples was used as the calibrator, and the results were expressed using the 2^−∆∆Ct^ method as a fold change relative to the controls. The primers sequences used are listed in Supplementary Table S1.

### 2.7. Malondialdehyde (MDA) Assay

Lipid peroxidation can be effectively detected by MDA evaluations. Therefore, we carried out MDA assays on colon tissues following the protocol outlined in previous studies [23].

### 2.8. ELISA Kits

ELISA kits were employed to measure the levels of 3-nitrotyrosine, glutathione (GSH), catalase (CAT), superoxide dismutase (SOD), iNOS, COX-2, Substance P, Claudin-1, ZO-1, Filaggrin, and Occludin in the esophagus, as well as the levels of TNF-α, IL-1β, IL-6, IL-8, PAF, reactive oxygen species (ROS), and reactive nitrogen species (RNS) in mice serums. For each kit, we followed the standard procedures as previously described [24], fully adhering to the manufacturer’s protocols.

### 2.9. Statistical Analysis

All experimental data in this study are presented as the mean ± standard deviation (SD) of N observations, where N denotes the number of animals. Data were first assessed for normal distribution and then analyzed using One-Way or Two-Way ANOVA, followed by Bonferroni correction conducted with GraphPad version 9.0 (La Jolla, CA, USA).

## 3. Results

### 3.1. Bombesin Reduces Epigastric Pain Induced by GERD

**Figure 1:**
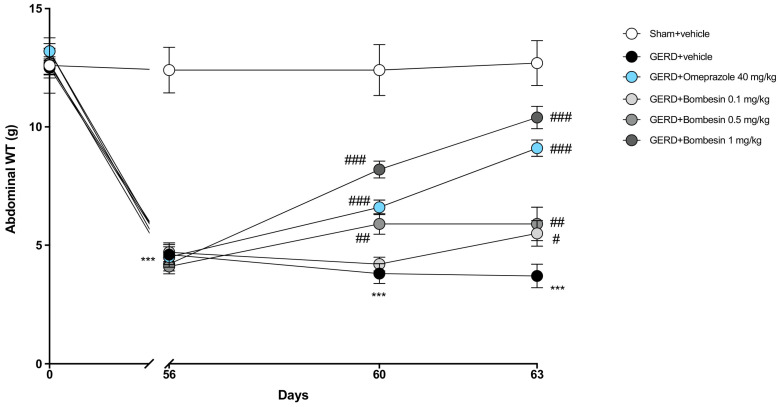
Effect of bombesin on epigastric pain induced by GERD. Von Frey test performed at different times in each experimental group: Sham + vehicle, GERD + vehicle, GERD + omeprazole 40 mg/kg, GERD + bombesin 0.1 mg/kg, GERD + bombesin 0.5 mg/kg and GERD + bombesin 1 mg/kg. In every experimental group, the number of mice was n = 8. Values are the means ± SD. Two-Way ANOVA test. *** *p* < 0.001 vs. Sham + vehicle group; # *p* < 0.05 vs. GERD + vehicle group; ## *p* < 0.01 vs. GERD + vehicle group; ### *p* < 0.001 vs. GERD + vehicle group.

The assessment of gastric pain linked to gastroesophageal reflux was performed by applying von Frey filaments to the epigastric region of each mouse to detect the mechanical stimulus–response. At day 0 (start of GERD induction), any significant change was found among all the experimental groups (Figure 1). Instead, at day 56 (GERD establishment), the GERD + vehicle group was characterized by a marked decrease in withdrawal threshold values compared to the Sham + vehicle group (Figure 1), showing epigastric hypersensitivity. After 4 days of oral treatments with bombesin and omeprazole (positive control group) (day 60), both compounds were able to significantly decrease epigastric pain compared to the GERD + vehicle group (Figure 1). At day 63, after 7 days of oral administrations, both compounds reduced abdominal pain compared to the GERD group (Figure 1).

### 3.2. Bombesin Modulates Gastric pH, MUC5AC, and MUC5B Levels

**Figure 2:**
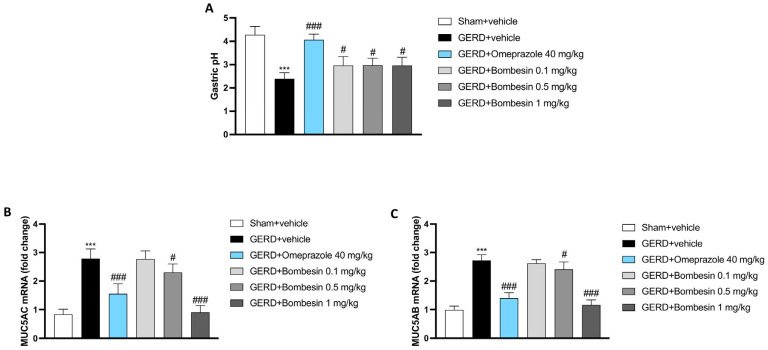
Effect of bombesin on the gastric pH, MUC5AC, and MUC5B levels. pH detected on gastric secretions with a microelectrode for each experimental group (**A**). RT-qPCR performed for the MUC5AC and MUC5AB levels in each experimental group (**B**,**C**). In every experimental group, the number of mice was n = 8. Values are the means ± SD. One-Way ANOVA test. *** *p* < 0.001 vs. Sham + vehicle group; # *p* < 0.05 vs. GERD + vehicle group; ### *p* < 0.001 vs. GERD + vehicle group.

Gastric pH was evaluated using a pH microelectrode. As shown in Figure 2A, the GERD group demonstrated a significant decrease of gastric pH compared to the Sham + vehicle group; however, the treatment with bombesin significantly re-established gastric pH in a dose-dependent manner, also compared to the omeprazole group (positive control) (Figure 2A).

Moreover, we decided to evaluate the levels of MUC5AC and MUC5AB, two important components of the mucus layer, by RT-qPCR. The GERD + vehicle group was characterized by an increase in the MUC5AC and MUC5AB levels compared to the Sham + vehicle group; however, bombesin treatments only at doses of 0.5 and 1 mg/kg significantly decreased their levels, overlapping with omeprazole administration (positive control) (Figure 2B,C).

### 3.3. Bombesin Reduces iNOS, COX2, 3-Nitrotyrosine, and Substance P Levels in the Esophagus

The levels of iNOS, COX-2, 3-nitrotyrosine, and substance P, important inflammatory mediators involved in GERD, were evaluated in the esophagus samples by RT-qPCR or the ELISA kit.

**Figure 3:**
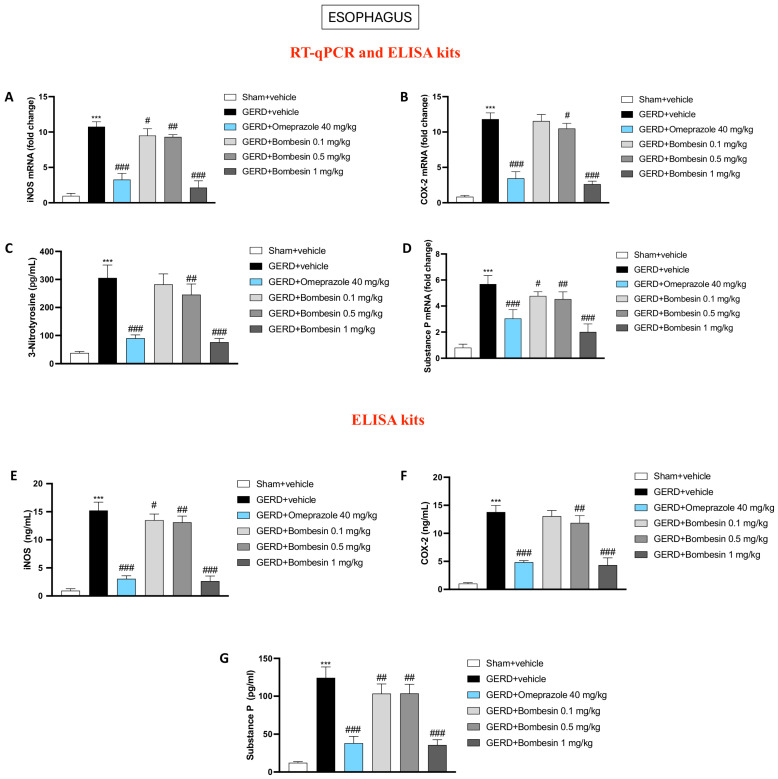
Effect of bombesin on the iNOS, COX2, 3-nitrotyrosine, and substance P esophageal levels. RT-qPCR and ELISA kits were performed for the detection of the following markers: iNOS (**A**–**E**), COX-2 (**B**–**F**), and Substance P (**D**–**G**). ELISA kit was performed to assess the levels of 3-nitrotyrosine in the esophagus (**C**). In every experimental group, the number of mice was n = 8. Values are the means ± SD. One-Way ANOVA test. *** *p* < 0.001 vs. Sham + vehicle group; # *p* < 0.05 vs. GERD + vehicle group; ## p < 0.01 vs. GERD + vehicle group; ### *p* < 0.001 vs. GERD + vehicle group.

Our data demonstrated that the GERD + vehicle group was characterized by a significant increase of the iNOS, COX2, 3-nitrotyrosine, and substance P levels compared to the Sham + vehicle group (Figure 3A–D); nonetheless, the treatment with bombesin, mainly at doses of 0.5 and 1 mg/kg, was able to decrease their levels (Figure 3A–D). Bombesin administration at the dose of 0.1 mg/kg was effective only in lessening iNOS and Substance P (Figure 3A–D).

As illustrated in Figure 3, the RT-qPCR results for iNOS, COX2, and substance P were validated also at the protein level using ELISA kits (Figure 3E–G).

### 3.4. Bombesin Modulates MDA, GSH, CAT, and SOD Levels in the Esophagus

**Figure 4:**
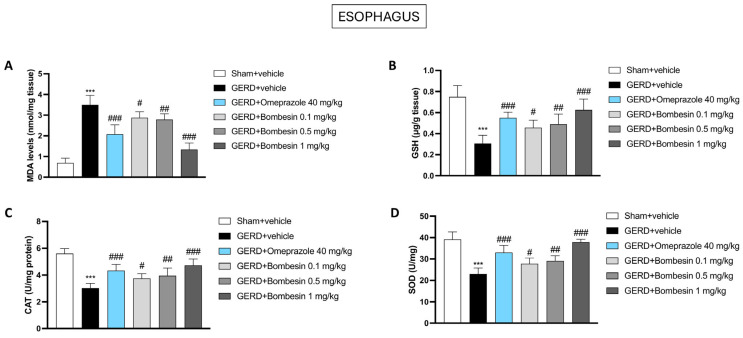
Effect of bombesin on the MDA, GSH, CAT, and SOD esophageal levels. Assays analyses of MDA (**A**), GSH (**B**), CAT (**C**), and SOD (**D**) for each experimental group. In every experimental group, the number of mice was n = 8. Values are the means ± SD. One-Way ANOVA test. *** *p* < 0.001 vs. Sham + vehicle group; # *p* < 0.05 vs. GERD + vehicle group; ## *p* < 0.01 vs. GERD + vehicle group; ### *p* < 0.001 vs. GERD + vehicle group.

To evaluate the oxidant–antioxidant endogenous balance following bombesin administration, we focused on reliable markers like MDA, GSH, CAT, and SOD, which were evaluated in the esophageal samples by several assays. Our data revealed a marked increase of the MDA levels and a significant decrease of the enzymatic antioxidant GSH, CAT, and SOD levels in the GERD + vehicle group compared to the Sham + vehicle group (Figure 4A–D). Nonetheless, the treatment with bombesin at doses of 0.1, 0.5, and 1 mg/kg was able to significantly decrease the MDA levels and re-establish the GSH, CAT, and SOD levels, as shown in Figure 4A–D.

### 3.5. Bombesin Re-Establishes the Claudin-1, ZO-1, Filaggrin, and Occludin Levels in the Esophagus

**Figure 5:**
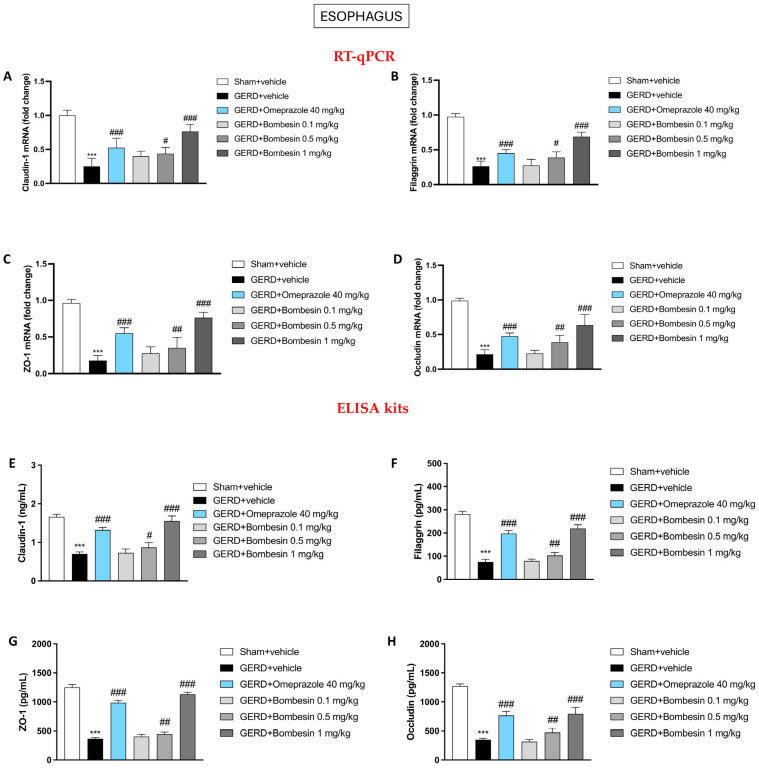
Effect of bombesin on the claudin-1, ZO-1, filaggrin, and occludin esophageal levels. RT-qPCR and ELISA kits were performed for the assessment of these TJs: claudin-1 (**A**–**E**), filaggrin (**B**–**F**), ZO-1 (**C**–**G**), and occludin (**D**–**H**). In every experimental group, the number of mice was n = 8. Values are the means ± SD. One-Way ANOVA test. *** *p* < 0.001 vs. Sham + vehicle group; # *p* < 0.05 vs. GERD + vehicle group; ## *p* < 0.01 vs. GERD + vehicle group; ### *p* < 0.001 vs. GERD + vehicle group.

GERD is characterized by an impaired epithelial barrier function that is regulated by cell–cell contact [25]. Therefore, we decided to evaluate the claudin-1, ZO-1, filaggrin, and occludin levels, important components of TJs in the esophagus samples, through the employment of RT-qPCR. Our results demonstrated a decrease of the claudin-1, ZO-1, filaggrin, and occludin levels in the GERD + vehicle group compared to the Sham + vehicle group (Figure 5A–D); however, following bombesin treatment at the highest doses of 0.5 mg/kg and 1 mg/kg, we observed a considerable re-establishment of the TJ levels (Figure 5A–D).

RT-qPCR data were confirmed by ELISA kits, as shown in Figure 5E–H.

### 3.6. Bombesin Reduces the TNF-α, IL-1β, IL-6, and IL-8 Levels in Serum

**Figure 6:**
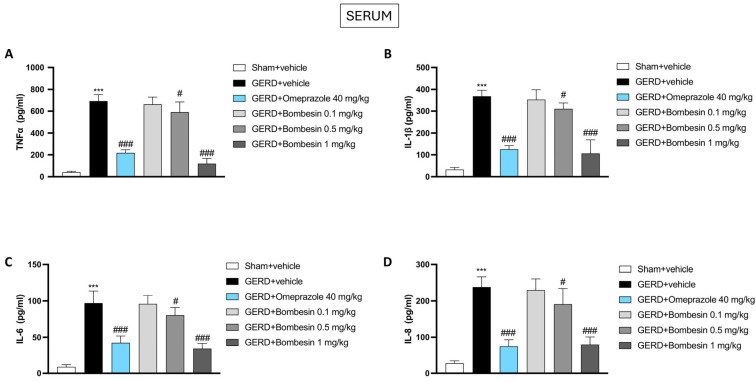
Effect of bombesin on the TNF-α, IL-1β, IL-6, and IL-8 levels in serum. ELISA kits performed on mice serum for the following inflammatory markers: TNF-α (**A**), IL-1β (**B**), IL-6 (**C**), and IL-8 (**D**). In every experimental group, the number of mice was n = 8. Values are the means ± SD. One-Way ANOVA test. *** *p* < 0.001 vs. Sham + vehicle group; # *p* < 0.05 vs. GERD + vehicle group; ### *p* < 0.001 vs. GERD + vehicle group.

To prove the anti-inflammatory effects of bombesin, the levels of proinflammatory cytokines such as TNF-α, IL-1β, IL-6, and IL-8 were evaluated in serum samples by an ELISA kit. The GERD + vehicle group was characterized by an increase of the TNF-α, IL-1β, IL-6, and IL-8 levels compared to the sham + vehicle group (*** *p*  <  0.001); however, the treatment with bombesin at doses of 0.5 and 1 mg/kg significantly reduced their levels, as shown in Figure 6A–D (# *p*  <  0.05; ### *p * <  0.001).

### 3.7. Bombesin Decreases the PAF, ROS, and RNS Levels in Serum

**Figure 7:**
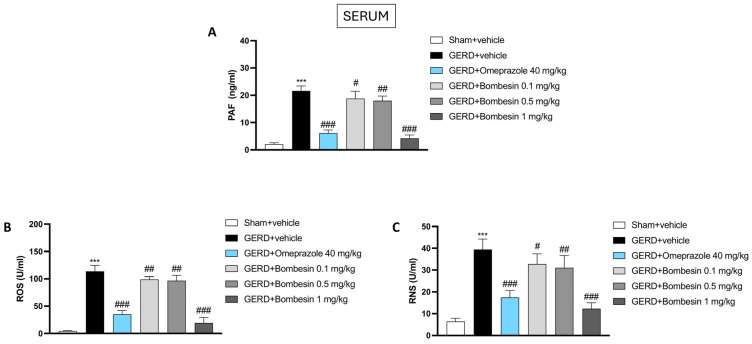
Effect of bombesin on the PAF, ROS, and RNS levels in serum. Serum levels of the following markers: PAF (**A**), ROS (**B**), and RNS (**C**) assessed by ELISA kits. In every experimental group, the number of mice was n = 8. Values are the means ± SD. One-Way ANOVA test. *** *p* < 0.001 vs. Sham + vehicle group; # *p* < 0.05 vs. GERD + vehicle group; ## *p* < 0.01 vs. GERD + vehicle group; ### *p* < 0.001 vs. GERD + vehicle group.

PAF, ROS, and RNS are important inflammatory and oxidative markers of GERD. In line with this, our data demonstrated that the GERD group was characterized by an increase in the PAF, ROS, and RNS levels compared to the serum levels of the Sham + vehicle group (Figure 7A–C); however, their levels were significantly reduced following the treatment with bombesin at doses of 0.1, 0.5, and 1 mg/kg, as shown in Figure 7A–C.

### 3.8. Bombesin Re-Establishes the Vitamin A, Vitamin B12, Vitamin C, and Vitamin E Levels in Serum

The vitamin levels are continuously monitored during long-term GERD therapies, since a gastrointestinal dyshomeostasis, together with commonly used therapies such as PPIs, can affect their absorption. Therefore, we decided to assess the vitamin A, vitamin B12, vitamin C, and vitamin E levels in serum samples using ELISA kits.

**Figure 8:**
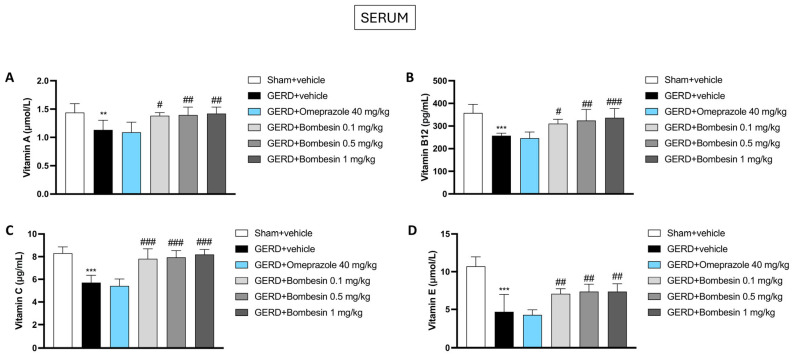
Effect of bombesin on the vitamin A, vitamin B12, vitamin C, and vitamin E levels in serum. ELISA kits performed to evaluate the levels of the following vitamins: vitamin A (**A**), vitamin B12 (**B**), vitamin C (**C**), and vitamin E (**D**). In every experimental group, the number of mice was n = 8. Values are the means ± SD. One-Way ANOVA test. ** *p* < 0.01 vs. Sham + vehicle group; *** *p* < 0.001 vs. Sham + vehicle group; # *p* < 0.05 vs. GERD + vehicle group; ## *p* < 0.01 vs. GERD + vehicle group; ### *p* < 0.001 vs. GERD + vehicle group.

Our results demonstrated that the GERD group was characterized by a decrease in the vitamin A, vitamin B12, vitamin C, and vitamin E levels compared to the Sham + vehicle group (Figure 8A–D). Following omeprazole 40 mg/kg treatment, we detected low levels of all analyzed vitamins compared to the GERD + vehicle group, confirming the influence of this therapy on worsening vitamin deficits.

However, the treatment with bombesin at doses of 0.1, 0.5, and 1 mg/kg significantly re-established the vitamin levels, demonstrating that it does not affect their absorption (Figure 8A–D).

## 4. Discussion

The need for discovering new therapeutic strategies for GERD is critical, as a significant subset of patients do not respond to conventional treatments. Current therapies, such as PPIs and H2 receptor antagonists, often fail to provide adequate relief for all sufferers [26,27]. This therapeutic gap underscores the importance of innovative research to develop more effective and comprehensive treatments. New approaches could target underlying mechanisms, improve symptom management, and enhance the quality of life for those with refractory GERD.

This is an important goal for research because of the high global incidence of GERD, which leads to significant health burdens and a higher risk of complications associated with the disease.

Previous studies performed by Tsai et al. have demonstrated that bombesin induces lower esophageal sphincter (LES) contraction via the BB2 and BB3 receptors [16]. BB2-mediated contraction results from extracellular Ca^2+^ influx through L-type Ca^2+^ channels, independent of nerve and muscarinic receptors, while BB3-mediated contraction involves neuronal conduction [16]. These findings suggested bombesin and BB2/BB3 agonists as potential GERD treatments, forming the basis for further in-depth experiments aiming to uncover the underlying mechanisms and the therapeutic application of this peptide.

Thus, by investigating the biological effects on inflammation, oxidative stress, and biochemical parameters, this research seeks to fully explore bombesin’s potential in treating GERD.

The reflux of gastric acid into the esophagus irritates the esophageal lining, leading to a burning sensation or discomfort in the upper abdomen [28]. Additionally, esophageal hypersensitivity and visceral hypersensitivity may contribute to the perception of pain, even without significant acid exposure [29]. Studies have highlighted that GERD-related abdominal pain often correlates with the severity of acid reflux and the presence of esophageal mucosal injury [30,31]. As a result of these harmful processes, the gastric discomfort typically occurs post-meal and can persist for hours, significantly impacting sleep quality and subsequently interfering with patients’ daily routines [32].

In the present study, the development of a GERD condition resulted in pronounced hypersensitivity in the upper abdomen, leading to heightened abdominal pain and responsiveness. However, administering bombesin effectively attenuated the increase in gastric sensitivity induced by GERD, demonstrating its ability to provide rapid relief of the symptoms as early as 4 days into treatment and more consistently after 7 days. In particular, at the dosage of 1 mg/kg, bombesin showed superior efficacy compared to omeprazole in alleviating this symptom.

Gastric acidity plays a central role in GERD, as the backflow of stomach acid into the esophagus irritates the esophageal lining. This acid reflux leads to symptoms such as heartburn, chest pain, and regurgitation. Persistent exposure to gastric acid can cause esophagitis, strictures, and even Barrett’s esophagus, a precursor to esophageal cancer. Thus, managing gastric acidity through lifestyle changes and appropriate medical care is crucial for alleviating symptoms and preventing complications associated with GERD.

In this study, mice with GERD showed a significant reduction in the pH levels of gastric secretions. Conversely, after one week of bombesin treatment, there was an appreciable modulation of intragastric pH, and this effect was likely related to the ability of this peptide to influence gastric motility.

In GERD patients, excessive or abnormal mucus production can have harmful effects [33]. Indeed, the overproduction of mucins like MUC5AC and MUC5AB in response to chronic acid exposure can lead to mucus hypersecretion, contributing to symptoms like coughing and throat clearing [34,35]. MUC5AC and MUC5AB present in both the stomach and esophagus can become dysregulated, exacerbating inflammation and impairing normal esophageal function [36,37]. This mucus overproduction can also create a barrier to the effective clearance of reflux acid, worsening the GERD symptoms and complicating the overall management of the disease [34,35].

In accordance, our data indicated a noteworthy upsurge of MUC5AC MUC5AB expression in esophageal tissues of GERD mice, which was considerably decreased following 7 days of administration of bombesin.

A complex interplay between inflammation, oxidative/nitrosative stress, and the neuropeptide substance P contributes to esophageal damage and symptom severity during GERD [38,39,40]. Chronic acid exposure triggers an inflammatory response in the esophageal mucosa, leading to the release of proinflammatory cytokines and recruitment of immune cells [41]. This inflammation is further amplified by oxidative and nitrosative stress, characterized by an excess of reactive oxygen and nitrogen species that damage the cellular components and exacerbate tissue injury [42]. Furthermore, substance P, a neuropeptide involved in pain transmission and inflammation, is upregulated in response to esophageal irritation and contributes to the sensation of pain and discomfort [40]. It also promotes the release of additional inflammatory mediators, perpetuating a cycle of inflammation and tissue damage [11]. The cooperation between these factors results in heightened sensitivity, mucosal erosion, and impaired healing, emphasizing the need for therapeutic strategies that target not only acid suppression but also inflammation and oxidative stress in GERD management.

From our data, it can be assumed that bombesin treatment was able to manage the local inflammatory state and oxidative/nitrosative stress by decreasing iNOS, COX-2, 3-nitrotyrosine, and substance P expression, proving to be an excellent therapeutic alternative to omeprazole.

As stated, the chronic exposure to gastric acid leads to an increase in ROS in the esophageal tissue, overwhelming the body’s natural antioxidant systems and impairing its homeostasis [43]. The reduced antioxidant defenses in GERD patients highlight the importance of enhancing the antioxidant capacity, either through diet, supplements, or targeted therapies, to mitigate oxidative damage and improve esophageal health [44].

The results from this study highlighted a noticeable decrease in the antioxidant capacities in GERD mice, which was effectively boosted by bombesin administration, as evidenced by the modulation of MDA, GSH, CAT, and SOD markers.

TJs, which are crucial for maintaining the integrity of the epithelial barrier, become disrupted due to chronic acid exposure [45]. This disruption increases the permeability of the esophageal lining, allowing acid and other irritants to penetrate deeper into the tissue, exacerbating inflammation and injury [45]. The loss of TJ integrity not only worsens GERD symptoms but also impedes the healing process.

In this regard, our results showed that, following the decrease in inflammation and oxidative/nitrosative stress, bombesin treatment also led to a restoration of the TJ levels, such as claudin-1, filaggrin, ZO-1, and occludin, that were previously altered by GERD establishment.

The inflammatory response initiated in the esophageal tissue during GERD can extend beyond the local environment and impact systemic circulation [46]. Chronic exposure to gastric acid induces persistent inflammation in the esophageal mucosa, leading to the release of proinflammatory cytokines such as IL-6, TNF-α, and IL-1β, and these cytokines can enter the bloodstream, promoting a systemic inflammatory state [46].

In fact, it has been demonstrated that the continuous release of these mediators not only exacerbates local esophageal injuries but also has potential implications for systemic health, contributing to conditions such as cardiovascular disease and metabolic syndrome [47,48]. Moreover, the systemic spread of inflammation can impair the body’s overall immune response, potentially leading to increased susceptibility to infections and other inflammatory conditions [49].

Our data confirmed a marked increase in cytokines and oxidative/nitrosative species in the systemic circulation following GERD; however, all these relevant markers were considerably decreased following bombesin administration.

PAF is a potent phospholipid activator involved in various inflammatory responses, including GERD [50]. In particular, when the PAF levels are elevated, there is an increased vascular permeability, recruitment of inflammatory cells, and release of proinflammatory cytokines [51]. This biological process exacerbates esophageal inflammation and tissue damage, contributing to symptoms such as pain and discomfort [52].

Here, we found that PAF was markedly increased after the induction of GERD; nevertheless, the administration of bombesin, mediating inflammatory processes, reduced the release of this inflammatory player, thus revealing once again powerful anti-inflammatory properties.

Medications commonly used to treat GERD, such as PPIs and H2 receptor antagonists, can lead to vitamin deficiencies over long-term use [53]. PPIs, by significantly reducing stomach acid production, can impair the absorption of essential vitamins and minerals [53]. For instance, reduced gastric acidity affects the absorption of vitamin B12, potentially leading to deficiency and associated issues such as anemia and neurological problems [54,55]. Similarly, the absorption of magnesium, calcium, and iron can be compromised, increasing the risk of osteoporosis and fractures [56]. H2 receptor antagonists, while less potent than PPIs, can still impact nutrient absorption over prolonged use [57]. Therefore, discovering new therapies that do not affect vitamin deficiencies could be crucial in patients undergoing long-term GERD treatment to prevent related health complications.

In this study, our findings suggested a positive correlation between bombesin administration and the reestablishment of vitamins levels. Since there is no direct evidence that bombesin influences vitamin levels, we assume that the restoration of gastrointestinal homeostasis exerted by the metabolic action of bombesin has consequently favored vitamin absorption and upsurge.

## 5. Conclusions

In conclusion, taking into consideration the obtained data, bombesin may represent a new and alternative therapeutic strategy for GERD management due to its ability to restore gastrointestinal homeostasis and motility. Thanks to these effects, abdominal pain, as well as inflammatory and oxidative/nitrosative markers, were modulated.

Bombesin has been shown to stimulate gastric emptying, potentially reducing the volume of gastric contents available for reflux into the esophagus. As proved by our study, this mechanism might help alleviate symptoms of GERD, also reducing the likelihood of acid reflux. However, one must be aware of the link between bombesin and gastrin, also in relation to individual variability, whereby responses to bombesin treatment may vary among individuals and factors such as age, underlying health conditions, and genetic variability could affect its efficacy and safety.

Therefore, despite these promising data, we must consider the limits of preclinical models, and thus, more studies are needed to better assess the effects of bombesin, for instance, in co-administration with omeprazole, as well as considering its evaluation in clinical investigations.

## Data Availability

All data in this study are included in this published article, and they are available at the corresponding author’s email.

## Supplementary Materials

The following supporting information can be downloaded at https://www.mdpi.com/article/10.3390/antiox13091043/s1: Table S1: Primer sequences used for real-time quantitative reverse transcriptase polymerase chain reaction (RT-qPCR).
