# Supplementary material for: Linking GERD and the Peptide Bombesin: A New Therapeutic Strategy to Modulate Inflammatory, Oxidative Stress and Clinical Biochemistry Parameters
Source: Antioxidants (Basel). 2024 Aug 28;13(9):1043. doi: 10.3390/antiox13091043 (PMC11428475; doi:10.3390/antiox13091043)
Supplement: Supplementary file 1 [file antioxidants-13-01043-s001.zip › antioxidants-3138424-supplementary.pdf]

Supplementary Table S1. Primer sequences used for real-time quantitative reverse transcriptase polymerase chain reaction (RT-qPCR)

Gene	Sequences	Primers
iNOS	Forward (5'-3')	GACATTACGACCCCTCCCAC
	Reverse (5'-3')	GCACATGCAAGGAAGGGAAC
COX-2	Forward (5'-3')	TTGGAGGCGAAGTGGGTTTT
	Reverse (5'-3')	TGGCTGTTTTGGTAGGCTGT
Substance P	Forward (5'-3')	TGGACATGGCCAGATCTCTCACAA
	Reverse (5'-3')	GCATCGCGCTTCTTTCATAAGCCA
Claudin-1	Forward (5'-3')	GTTTGCAGAGACCCCATCAC
	Reverse (5'-3')	AGAAGCCAGGATGAAACCCA
Filaggrin	Forward (5'-3')	CTTCTGGAAGGA CAACTACAGGC
	Reverse (5'-3')	GCTTTTGCCAGCTTTAGCACCAG
ZO-1	Forward (5'-3')	ACAGGCCATTACGAGCCTCT
	Reverse (5'-3')	GGAGGCTGTGGTTTGGTAGC
Occludin	Forward (5'-3')	CTCCCATCCGAGTTTCAGGT
	Reverse (5'-3')	GCTGTCGCCTAAGGAAAGAG
β-actin	Forward (5'-3')	TGCTGTCCCTGTATGCCTCTG
	Reverse (5'-3')	TGATGTCACGCACGATTTCC
